# Rotational stability of the AcrySof SA60TT toric intraocular lenses: A cohort study

**DOI:** 10.1186/1471-2415-8-8

**Published:** 2008-05-06

**Authors:** Bruno Zuberbuhler, Theo Signer, Richard Gale, Eduard Haefliger

**Affiliations:** 1 Moorfields Eye Hospital NHS Foundation Trust, 162 City Road, London, EC1V 2PD, UK; 2 Vista Klinik, Hauptstrasse 55, CH-4102 Binningen, Switzerland; 3 St Thomas' Hospital, Ophthalmology Department, London, SE1 7EH, UK

## Abstract

**Background:**

To evaluate the rotational stability of the three types of AcrySof SA60TT toric intraocular lenses (Alcon, Switzerland) in cataract surgery after the first postoperative week.

**Methods:**

A retrospective study of 44 eyes in 33 patients. All patients underwent similar uncomplicated phacoemulsification cataract surgery. Seven eyes with corneal astigmatism of less than 1.5 D were implanted with the AcrySof SA60T3 intraocular lens. Seventeen eyes with astigmatism between 1.5 D and 2.25 D received the SA60T4 intraocular lens, and 20 eyes with more than 2.25 D of corneal astigmatism received the SA60T5 intraocular lens. Intraoperatively, the axis of the toric lens was aligned to the steepest axis of the corneal astigmatism. Main outcome measure was the postoperative position of the lens, assessed at 1 week and 3 months, using a specially designed angle measuring eyepiece for the slit lamp.

**Results:**

There was no significant difference in the rotational stability of the three types of toric intraocular lenses. Overall, the postoperative rotation was within 5 degrees in 95% and within 2 degrees in 68% of eyes. The mean absolute rotation was 2.2 ± 2.2 degrees. No lens showed more than 9 degrees of rotation, and no lens required secondary repositioning. There was no trend for either clockwise or anti-clockwise rotation. The surgical procedure did not change the corneal astigmatism.

**Conclusion:**

Once placed to it's position, each of the three types of the AcrySof SA60TT toric intraocular lenses demonstrate rotational stability in the capsular bag.

## Background

Corneal astigmatism above 1.5 D occurs in 19% of patients [1]. Implanting a toric intraocular lens (IOL) offers the possibility of correcting not only spherical equivalent refraction, but also astigmatism during phacoemulsification cataract surgery. The AcrySof SA60TT toric series of IOLs (Alcon, Switzerland) is made of hydrophobic Acrylate and shares the same biconvex single-piece design as the AcrySof SA60AT monofocal IOL (Alcon, Switzerland). The toric IOL differs in that there is a toric component located on the posterior surface of the lens optic. The optic is marked with 3 peripheral dots that indicate the cylindrical axis of the lens and so enables its correct alignment with the steepest axis of the corneal astigmatism during surgery. The AcrySof toric IOL is available in 3 types with different cylinder powers: 1.5 D (SA60T3), 2.25 D (SA60T4) and 3.0 D (SA60T5).

The success of a toric IOL can be judged not only by its ability to reduce refractive astigmatism immediately postoperatively, but also by its ability to maintain a stable position in the capsular bag in the longer term. However, many surgeons have stopped using toric IOLs because of reported positional instability [2]. The most frequent cause of IOL rotation following uncomplicated cataract surgery is because of capsular bag shrinkage due to fibrosis [3]. The majority of this occurs within the first three months after implantation [4]. Even a small rotational deviation of the toric IOL from its intended axis can result in large reduction of the astigmatic correction [5]. For example, a deviation of 10 degrees minimizes the potential correction by 35%.

The aim of this study was to evaluate the postoperative rotational stability of the three types of AcrySof SA60TT toric IOLs in standard cataract surgery between the first postoperative week and the third month.

## Methods

This is a retrospective cohort study of 44 eyes of 33 patients. The eyes all had corneal astigmatism of 1 to 5 D, and between July 2006 and March 2007 received an AcrySof toric intraocular lens during cataract surgery. Exclusion criteria were axial length of less than 22 mm, zonular weakness, a change in refractive astigmatism of more than 0.5 D within the 3 months prior to surgery, and coexisting maculopathy or glaucoma.

Eyes with a corneal astigmatism (Orbscan, Carl Zeiss, Germany) of less than 1.5 D were implanted with the AcrySof SA60T3 IOL, eyes with a corneal astigmatism between 1.5 D and 2.25D were implanted with the SA60T4 IOL, and eyes showing a corneal astigmatism of more than 2.25 D were implanted with the SA60T5 IOL. A total of 44 eyes of 33 patients were recruited.

Biometry was performed with the IOL Master (Carl Zeiss, Germany), using the Haigis formula for the IOL power calculation and the company's recommended A-constant of 118.4 for the toric IOL. The target postoperative spherical equivalent was aimed to emmetropia.

Preoperatively, the steepest axis of the corneal astigmatism, determined by Orbscan topography was marked at the corneal limbus with a marker pen, using an angle measuring eyepiece on a BQ900 slit lamp (Haag Streit, Switzerland). Cataract surgery was performed by a single surgeon (EH) at the Vista Klinik, Binningen, Switzerland under topical local anesthesia. Each patient underwent the same technique with a 2.8 mm superior clear limbal incision, continuous curvilinear capsulorhexis, phacoemulsification with cleaver technique and bimanual irrigation/aspiration. The toric IOL was implanted into the capsular bag with a Monarch II injector and a B-Cartridge. Subsequently, the IOL was rotated with a second instrument, so that the cylindrical axis of the lens was aligned with the limbal marks of the corneal astigmatism. All patients were prescribed a combination of tobramicin and dexamethasone eye drops 4 times daily for 4 weeks.

At 1 week and 3 months postoperatively all patients underwent best-corrected distance visual acuity, subjective refraction and slit lamp examination. The postoperative corneal astigmatism was assessed by Orbscan topography at 3 months.

For the measurement of the axis of the toric lens the pupil was dilated to enable the peripheral dots on the optic to become visible. A specially designed eyepiece, replacing one of the 10× eyepieces, was inserted in the slit lamp. Looking through the eyepiece, a spirit level, a horizontal line, a black measurement line and a circular scale in the periphery with single degree steps could be seen. With the spirit level the horizontal line could be adjusted to the horizontal axis. A virtual reference line was used, formed by the line between the pupils of patient's eyes, to provide a patient's reference. This reference line was aligned with the horizontal line of the eyepiece at each follow-up to guarantee reproducible measuring conditions. The black measurement line then could be rotated into the axis of the toric IOL. The angle between the black measurement line and the horizontal line represented the angle of the toric IOL. The angle could be read on the peripheral scale in degrees.

Statistical analysis was performed using SPSS Software (SPSS Inc., Chicago, IL, USA). For comparative statistics the Wilcoxon matched-pairs test was used and for independent samples and the Mann-Whitney U-test was used. For association statistics the Spearman rank correlation test was applied. P-values less than 0.05 were considered statistically significant.

## Results

Forty-four eyes of 33 patients underwent routine phacoemulsification cataract surgery with in-the-bag implantation of a toric AcrySof IOLs. Seven eyes were implanted with the SA60T3 IOL, 17 eyes were implanted with the SA60T4 IOL, and 20 eyes were implanted with the SA60T5 lens (Table 1). There was no difference seen in patients' age, preoperative best-corrected visual acuity and preoperative spherical equivalent between the three types of IOLs.

**Table 1:** Patient demographics, best-corrected distance visual acuity, refractive astigmatism and corneal astigmatism before and three months after implantation of a AcrySof SA60TT toric intraocular lens (SA60T3, SA60T4 or SA60T5 type).

Parameters	SA60T3	SA60T4	SA60T5	Summarized
Eyes (n)	7	17	20	44
Patients (n)	7	14	17	33
Age (y)				
Mean ± SD	66 ± 9	71 ± 14	72 ± 13	71 ± 13
Range	52 to 76	35 to 88	35 to 86	35 to 88
Preoperative Parameters				
Mean BCDVA (LogMAR) ± SD	0.28 ± 0.22	0.27 ± 0.15	0.30 ± 0.17	0.28 ± 0.17
Mean SE (D) ± SD	-1.90 ± 3.27	-1.60 ± 3.38	-1.50 ± 3.15	-1.60 ± 3.19
Mean refractive astigmatism (D) ± SD*	1.86 ± 0.83	2.24 ± 0.83	2.98 ± 1.07	-
Mean corneal astigmatism (D) ± SD^†^	1.40 ± 0.55	1.95 ± 0.56	3.06 ± 0.74	-
Postoperative Parameters				
Mean BCDVA (LogMAR) ± SD	0.00 ± 0.06	-0.02 ± 0.04	0.05 ± 0.15	0.01 ± 0.11
Mean SE (D) ± SD	-0.75 ± 0.95	-0.80 ± 1.44	-0.38 ± 1.26	-0.61 ± 1.28
Mean refractive astigmatism (D) ± SD*	0.43 ± 0.59	0.22 ± 0.26	0.89 ± 1.29	-
Mean corneal astigmatism (D) ± SD^†^	1.54 ± 0.51	1.85 ± 0.50	3.19 ± 0.93	-

BCDVA = best-corrected distance visual acuity; SE = spherical equivalent

*The change in refractive astigmatism was statistically significant due to the lens implantation (SA60T3 p = 0.04; SA60T4 p < 0.001; SA60T5 p < 0.001).

^†^The corneal astigmatism did not change significantly due to the surgery (SA60T3 p = 0.17; SA60T4 p = 0.98; SA60T5 p = 0.26).

**Figure 1:**
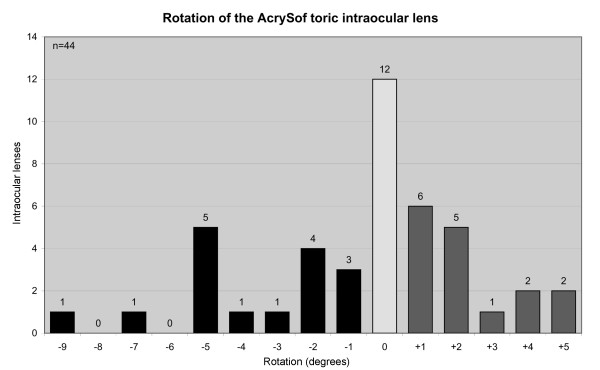
**Rotation of the AcrySof SA60TT toric intraocular lens between the one week and three months postoperative follow-up**.

There was no difference in rotational stability between the three IOL types (p > 0.18). Between week 1 and month 3 the overall mean absolute rotation of all 44 implanted IOLs was 2.2 ± 2.2 degrees. Sixteen (36%) IOLs rotated clockwise with a maximum rotation of 9 degrees, sixteen (36%) IOLs rotated anti-clockwise with a maximum rotation of 5 degrees, and twelve (28%) IOLs did not rotate (Figure 1). In 95% of eyes the IOL rotation was 5 degrees or less and in 68% of eyes it was 2 degrees or less. No correlation was seen between the amount of postoperative rotation and the topographic Sim K readings (p = 0.760), the axial length of the eye (p = 0.773) or the IOL power (p = 0.801).

The corneal astigmatism did not significantly change following surgery (p = 0.17 for SA60T3; p = 0.98 for SA60T4; p = 0.26 for SA60T5). The mean reduction in refractive astigmatism was 1.43 D for the SA60T3 IOL, 2.02D for the SA60T4 IOL, and 2.09D for the SA60T5 IOL. A reduction of at least 1 D was achieved in 71% of eyes receiving a SA60T3 IOL, a reduction of at least 1.5 D was achieved in 65% of eyes receiving a SA60T4 IOL, and a reduction of at least 2 D was achieved in 61% of eyes receiving a SA60T5 IOL. There was no correlation between the rotation of the lens and the amount or the change in refractive astigmatism (p = 0.931).

At three months, 90% of eyes were within 1.0 D and 81% of eyes within 0.5 D of the target spherical equivalent. Ninety-six percent of eyes gained one or more Snellen line of best-corrected distance visual acuity (BCDVA); 82% of eyes gained two or more Snellen lines of BCDVA. One eye lost one line of BCDVA.

No complication was encountered during the intra- and postoperative course. None of the lenses required secondary alignment.

## Discussion and Conclusion

This is the first study to report the rotational stability of the AcrySof SA60TT toric IOL in a series of 44 cases 3 months after implantation in cataract surgery.

The rotational stability of the SA60TT toric IOLs compares favorably with other current-generation toric IOLs. The SA60TT showed a mean absolute rotation of 2.2 degrees, a maximum rotation of 9 degrees and 95% of IOLs showed 5 degrees or less of rotation (Table 2). De Silva et al. [6] showed in a series of 21 MicroSil 6116TU toric IOLs with Z-haptics (HumanOptics, Germany) that the mean rotation of this lens was 5.2 degrees and the maximum rotation was 15 degrees. Only 60% of the IOLs demonstrated five degrees or less of rotation. Sixty percent of the MicroSil toric IOLs rotated anti-clockwise [7]. Chang [8] demonstrated in a series of 50 STAAR TL toric IOLs (STAAR, USA) a maximum rotation of 20 degrees and 72% of the IOLs were within 5 degrees of the intended axis. A smaller diameter version of this STAAR IOL (STAAR TF toric IOL) demonstrated rotation of up to 80 degrees and required subsequent repositioning in 50% of cases [8]. Other currently used toric IOLs include the T-flex 573T and T-flex 623T (Rayner, United Kingdom), and the Acri.LISA Toric 466TD and Acri.Comfort 646TLC (Acri.Tec, Germany).

**Table 2:** Absolute rotation and direction of rotation of the AcrySof SA60TT toric intraocular lenses between the one week and three months follow-up after implantation in standard cataract surgery.

Parameters	SA60T3	SA60T4	SA60T5	Summarized
Eyes (n)	7	17	20	44
Absolute rotation (degrees)				
Mean ± SD	1.6 ± 1.0*	3.3 ± 2.4*	1.5 ± 2.0*	2.2 ± 2.2
Range	0 to 3	0 to 9	0 to 7	0 to 9
Direction of rotation, n (%)				
Clockwise	4 (57%)	7 (41%)	5 (25%)	16 (36%)
Anti-clockwise	2 (29%)	7 (41%)	7 (35%)	16 (36%)
No rotation	1 (14%)	3 (18%)	8 (40%)	12 (28%)

*Statistically, no difference in absolute rotation was noticed between the three types (p > 0.18).

In this series, the largest rotation of the toric IOL was 9 degrees, seen in one eye only. No specific cause was identified for this large deviation and the patient did not require secondary repositioning of the lens because of visual satisfaction. One eye lost a single line of BCDVA because of a disadvantageous change in the regularity of the corneal astigmatism. No reason could be found for this anomaly. This patient was managed conservatively.

A toric lens has to be implanted using an astigmatically neutral procedure: a technique that will have no effect on the preoperative corneal astigmatism [5]. This study demonstrated no significant change in corneal astigmatism from the preoperative assessment to the 3 month postoperative assessment. Caution should be exercised while implanting and rotating the toric lens in the capsular bag so as not to cause capsular damage. Very small eyes, eyes with zonular instability or floppy capsules (post-vitrectomy eyes) may be considered a relative contraindication.

Except for the SA60T5, the AcrySof SA60TT lenses reduced refractive astigmatism as expected. The manufacturer's data claim that the cylinder power of the SA60T5 IOL is 0.75 diopters higher at the IOL plane, and 0.51 D higher at the corneal plane, than the SA60T4. However, the results of this study show a mean difference in refractive cylinder power between the two types of only 0.1 D. Theoretically, this could be due to errors in our refractive data. Further studies with a longer follow-up are necessary to analyze the refractive outcome of the toric IOLs.

Different methods can be used to accurately determine the position of a toric IOL. Weinand et al. [9] and Becker et al. [10] analyzed digital and conventional photographs, taken preoperative and postoperative through the slit lamp and operating microscope. This method with photographs in retrograde illumination has become the 'gold standard' for evaluating the centration and axial positioning of a toric intraocular lens. Viestenz et al. [11,12] and Quentin et al. [13] used the method of simultaneous slide projection to evaluate the rotation of the toric lens. This method has shown superior accuracy in calculating the rotational stability, because it respects the autorotation of the eye which has shown to be up to 11.5 degrees (mean of 2.3 degrees) [12,14]. The negative side of the methods with photographs or slides is the increased time consume and demand for specific equipment. In this study, we choose the eyepiece measuring device because it was only a postoperative analysis of the toric lens axis. The device was easy and fast to handle and allowed the ophthalmologist to perform axis measurements at any follow-up, even in busy clinics. It also showed good reproducibility when the patient's head was correctly aligned with the eyepiece and the slit lamp. Because the eyepiece provided data with one degree precision, this unit was used for the study, too. The precision of one degree steps, of course is on the optimistic side, and needs to be set in relation to the errors occurring from autorotation and head dislocation. For comparisons of misalignments of the toric lens, analyzing preoperative and postoperative axis, respecting parallaxes and cyclorotation, we recommend to use the superior technique of simultaneous slide projection.

In conclusion, the three types of AcrySof SA60TT toric IOLs demonstrated no significant rotation three months after implantation. The surgical technique used for implantation did not significantly change the axis of corneal astigmatism.

## Competing interests

The authors declare that they have no competing interests.

## Authors' contributions

BZ conceived of the study, participated in its design and coordination, collected data and wrote the article. RG performed the statistical analysis and contributed to writing the article. TS and EH conceived of the study, participated in its design and contributed to writing the article. All authors read and approved the final manuscript.

## Pre-publication history

The pre-publication history for this paper can be accessed here:



## References

[B1] Hoffer KJ (1980). Biometry of 7,500 cataractous eyes. Am J Ophthalmol.

[B2] Horn JD (2007). Status of toric intraocular lenses. Curr Opin Ophthalmol.

[B3] Ohmi S (1993). Decentration associated with asymmetric capsular shrinkage and intraocular lens size. J Cataract Refract Surg.

[B4] Strenn K, Menapace R, Vass C (1997). Capsular bag shrinkage after implantation of an open-loop silicone lens and a poly(methyl methacrylate) capsule tension ring. J Cataract Refract Surg.

[B5] Sanders DR, Grabow HB, Shepherd J, Gills JP, Martin RG, Sanders DR (1992). The toric IOL. Sutureless Cataract Surgery; An Evolution Toward Minimally Invasive Technique.

[B6] De Silva DJ, Ramkissoon YD, Bloom PA (2006). Evaluation of a toric intraocular lens with Z-haptic. J Cataract Refract Surg.

[B7] Warlo I, Krummenauer F, Dick HB (2005). Rotational stability in intraocular lenses with C-haptics versus Z-haptics in cataract surgery. A prospective randomised comparison. Ophthalmologe.

[B8] Chang DF (2003). Early rotational stability of the longer Staar toric intraocular lens: fifty consecutive cases. J Cataract Refract Surg.

[B9] Weinand F, Jung A, Stein A, Pfotzner A, Becker R, Pavlovic S (2007). Rotational stability of a single-piece hydrophobic acrylic intraocular lens: new method for high-precision rotation control. J Cataract Refract Surg.

[B10] Becker KA, Auffarth GU, Völcker HE (2004). Measurement method for the determination of rotation and decentration of intraocular lenses. Ophthalmologe.

[B11] Viestenz A, Langenbucher A, Seitz B (2006). Impact of the eye's cyclorotation on axial orientation analysis of toric intraocular lenses: recommendations for an optimized evaluation of rotational stability of toric IOLs. Klin Monatsbl Augenheilkd.

[B12] Viestenz A, Seitz B, Langenbucher A (2005). Evaluating the eye's rotational stability during standard photography. Effect on determining the axial orientation of toric intraocular lenses. J Cataract Refract Surg.

[B13] Quentin CD, Genée D, Auffarth GU, Welt R, Demeler U, Köln, Biermann Rotationsstabilität der Silikon-HKL mit C-Haptik versus Z-Haptik im Kapselsack und mit einer C-Haptik im Sulcus ciliaris. 17 Kongress der Deutschsprachigen Gesellschaft für Intraokularlinsen-Implantation und refraktiven Chirurgie.

[B14] Viestenz A, Walter S, Viestenz A, Behrens-Baumann W, Langenbucher A (2007). Torische Intraokularlinse und Astigmatismuskorrektur. Ophthalmologe.

